# Synergistic Effect of Viral Load and Alcohol Consumption on the Risk of Persistent High-Risk Human Papillomavirus Infection

**DOI:** 10.1371/journal.pone.0104374

**Published:** 2014-08-20

**Authors:** Hea Young Oh, Sang-Soo Seo, Mi Kyung Kim, Dong Ock Lee, Youn Kyung Chung, Myong Cheol Lim, Joo-Young Kim, Chan Wha Lee, Sang-Yoon Park

**Affiliations:** 1 Division of Cancer Epidemiology and Prevention, National Cancer Center, Goyang-si, Gyeonggi-do, Korea; 2 Center for Uterine Cancer, National Cancer Center, Goyang-si, Gyeonggi-do, Korea; 3 Center for Cancer Prevention and Detection, Hospital, National Cancer Center, Goyang-si, Gyeonggi-do, Korea; Georgetown University, United States of America

## Abstract

**Purpose:**

This prospective study aimed to examine the combined effect of viral load and alcohol consumption on the risk of persistent high-risk (HR) human papillomavirus (HPV) infection.

**Methods:**

Among women undergoing health screening between 2002 and 2011 at the National Cancer Center, 284 and 122 women with HR-HPV infection and cytological findings of low-grade squamous intraepithelial or lower-grade lesions were followed up for 1 and 2 years, respectively. Multivariate logistic regression analysis was performed, and the relative excess risk due to interaction (RERI) and synergy index (S) were calculated.

**Results:**

Among drinkers, the risks of 1-year (odds ratio [OR] 4.09, 95% confidence interval [CI] 2.05–8.18) and 2-year persistence (OR 8.08, CI 2.36–27.6) were significantly higher for high HPV loads than for low HPV loads; this association was not seen for non-drinkers. The risks for 1-year (OR 4.14, CI 1.89–9.05) and 2-year persistence (OR 6.61, CI 2.09–20.9) were significantly higher in subjects with a high HPV load who were also drinkers than in those who were non-drinkers. A high HPV load together with a longer drinking duration or higher alcohol consumption was associated with increased risks of 1-year (OR 3.07, CI 1.40–6.75 or OR 2.05, CI 0.87–4.83) and 2-year persistence (OR 6.40, CI 1.72–23.8 or OR 4.14, CI 1.18–14.6). The synergistic effect of alcohol consumption and HR-HPV load was stronger on the risk of 2-year persistence (RERI = 3.26, S = 2.38) than on the risk of 1-year persistence (RERI = 1.21, S = 1.63).

**Conclusions:**

The synergistic effect of HR-HPV load and alcohol consumption was associated with the risk of HR-HPV persistence and was stronger for longer-term HR-HPV infection. Limiting alcohol consumption might be an important measure to prevent the development of cervical cancer in women with a high HR-HPV load.

## Introduction

Persistent high-risk human papillomavirus (HR-HPV) infection is an important cause of cervical intraepithelial neoplasia (CIN) and cervical cancer [1]–[3]. HPV persistence is associated with virus-related factors such as viral genotype, multiplicity of infection, and viral load [4] as well as host-related factors, including old age [2], multiple lifetime sexual partners [5], cigarette smoking [5], compromised immune response [6], and oral contraceptive use [7].

Among these factors, the use of HR-HPV load as a marker for predictor of persistence remains controversial. Several studies have reported HPV-16 load to be associated with persistent infection [8], [9], cytological severity of cervical lesions [10], [11], and pre-cancerous or cancerous cervical lesions [8], [12]. However, some studies have shown HPV load to be of limited use as a clinical parameter to discriminate between lesion grades or to predict HPV persistence or CIN in young women with normal cytology or invasive cervical carcinoma disease progression [13]–[15].

Cigarette smoking and alcohol consumption are critical factors in carcinogenesis and immune suppression. Cigarette smoking is reported to be a critical risk factor for cervical cancer and its high-grade precursors [16]. Alcohol consumption is also associated with an increased risk of HPV infection, but reports on the association between alcohol consumption and the persistence of HPV infection are limited [2], [17]. To investigate the combined effects of viral load and cigarette smoking, two studies reported an association between cigarette smoking and HPV-16 DNA load in cervical carcinoma *in situ* (CIS) development and low-grade cytological abnormalities [18], [19]. However, to our knowledge, except for our previous study [20], research on the combined effect of HPV load and alcohol consumption on cervical cancer development has not been undertaken.

In this prospective study, we assessed the associations of HR-HPV load and alcohol consumption with persistent HR-HPV infection. We used health screening data to investigate the combined effect of HR-HPV load and alcohol consumption, with its different characteristics, on the risk of persistent HR-HPV infection.

## Materials and Methods

### Subjects and groups

This cohort study was part of the Korean Prospective Study for the Transition of Human Papillomavirus into Cervical Carcinoma (KOVIC). Among 11,140 Korean women undergoing health screening at the National Cancer Center between 2002 and 2011 who provided written informed consent, 920 were positive for HR-HPV on a DNA test at enrollment and responded to questions about alcohol consumption. Subjects receiving any therapy or surgery or using immunosuppressive agents were excluded at enrollment. Of these, 284 and 122 women with low-grade squamous intraepithelial lesions (LSIL) or lesions of lower cytological grade at enrollment were eligible for HR-HPV persistence follow-up studies 1 and 2 years after enrollment. Four women (3 for the 1-year study and 1 for the 2-year study) were excluded because of lesions with cytology grades greater than LSIL during the follow-up period. Among 284 women in the 1-year follow-up group, 122 with persistent HR-HPV infection (3 consecutive positive results for HPV DNA) at 2 years after enrollment were analyzed in the 2-year follow-up study. Subjects were divided into 2 groups, the clearance and persistence groups, and were analyzed in 1-year and 2-year follow-up studies (Table 1). Clearance was defined for both follow-up groups as HPV positivity at baseline with HPV negativity 1 year (n = 148) or 2 years later (n = 136) regardless of intermediate results. Persistence was defined as HPV positivity at baseline with HPV positivity at 1 (n = 66) or 2 years later (n = 56), regardless of intermediate results. Patient data collected included HR-HPV DNA status and viral load; Papanicolaou test (Pap smear) results; and comprehensive lifestyle questionnaire items including age, body mass index, marital status, menopausal status, oral contraceptive use, parity, education, alcohol consumption, and smoking habits.

**Table 1:** Definition of study groups for the persistence of high risk-human papillomavirus.

	HR-HPV statusduring studyyear	N	Enrolment	First studyyear afterenrollment	Second studyyear afterenrollment
1-year follow up (n = 284)	Clearance	148	+	−	
	Persistence	136	+	+	
2-year follow up (n = 122)	Clearance	66	+	− or +	−
	Persistence	56	+	− or +	+

Of 284 women positive for high-risk human papillomavirus (HR-HPV) and cytological findings of low-grade squamous intraepithelial or lower-grade lesions at enrollment who were evaluated at the 1-year follow-up assessment, 122 returned for the 2-year follow-up. Clearance in the 1-year follow-up group was defined as HPV positivity at baseline with HPV negativity after 1 year; similarly, persistence in the 1-year follow-up group was defined as HPV positivity both at baseline and 1 year later. Clearance and persistence of the 2-year follow-up group were defined as HPV positivity at baseline with HPV negativity and HPV positivity after 2 years, respectively, regardless of intermediate results. HR-HPV status was determined using the commercial Hybrid Capture 2 test.

### Ethics statement

The Institutional Review Boards and Ethics Committee of the National Cancer Center in Korea (NCCNCS-11-433) approved this study in March 2011 and written informed consent was obtained from all subjects.

### Questionnaires related to alcohol consumption

Detailed self-administered health and lifestyle questionnaires, including questions on behavior related to alcohol consumption, were completed at enrollment. Questions related to alcohol consumption were aimed at determining the alcohol consumption status for the previous 5 years (current, former, or never), the frequency of alcohol consumption (1 day/month, 2–3 days/month, 1 day/week, 2–3 days/week, 4–5 days/week, every day, or 2 times/day), the duration of the drinking habit (years), and the typical volume per drink (1 glass, 2 glasses, ≥3 glasses) of beer (200 mL) or soju (50 mL) for the previous 5 years. Daily alcohol consumption was calculated individually for frequency and typical volume per drink [21].

### HR-HPV DNA detection and Pap smear

HR-HPV DNA was detected using the commercially available Hybrid Capture 2 assay (HC2, Digene Co. Silver Spring, MD. USA). The chemiluminescent HPV DNA test yielded relative light units (RLU) using a probe designed to detect 13 HR-HPV types. Results were considered HR-HPV positive at concentrations of 1.20 pg/mL or greater than the RLU cut-off ratio (specimen RLU/mean RLU of 2 positive controls [PCs]) and borderline at concentrations of 0.80–1.20 pg/mL. Cervical cytological findings were classified using the Bethesda classification system.

### Statistical analysis

Descriptive statistics and logistic regression analyses were performed using SAS 9.1 (SAS Institute, Cary, NC, USA). Chi-square and *t*-tests were used to analyze distribution differences in categorical and continuous variables. The Wilcoxon rank sum test was used to analyze differences in raw viral loads. Multivariate logistic regression analysis was performed to evaluate the association between HR-HPV load (low: <100 RLU/PC and high: ≥100 RLU/PC), alcohol consumption (non-drinkers and drinkers), other variables related to alcohol consumption (duration and daily amount of alcohol consumption), and HPV persistence (1-year and 2-year persistence). Risk estimates were calculated with low HPV load, non-drinking status, alcohol consumption for <5 years, and alcohol consumption of <15 g alcohol/day as reference categories.

The risk of HR-HPV persistence was subsequently analyzed according to HPV load in non-drinkers and drinkers. We divided an ordered distribution of HPV load into tertiles, and risk estimates for 1-year and 2-year persistence were calculated with low viral load as reference. All variables were adjusted for age as a continuous variable and for menopausal status (pre, post), oral contraceptive use (never, past/current), smoking status (never, past/current), and number of children (none or 1, 2, ≥3) as categorical variables.

To assess the combined effect of HR-HPV load and alcohol consumption on the risk of persistent HR-HPV infection, interaction tests were performed on additive scale in the model for viral load and drinkers or other variables related to alcohol consumption. The relative excess risk due to interaction (RERI) and synergy index (S) with 95% confidence intervals and *p* values were calculated according to previously described methods for measuring biological interaction between risk factors [22], [23]. Odds ratio (OR) were adjusted for age alone or for age, menopausal status, oral contraceptive use, smoking status, and number of children using logistic regression analysis for drinkers with a high viral load, non-drinkers with a high viral load, and drinkers with a low viral load, compared to non-drinkers with a low viral load. RERI>0 and S>1.0 indicated a synergistic effect with a high viral load and alcohol consumption.

## Results

### Subject characteristics

Mean ages did not differ between the 1-year clearance and 1-year persistence groups (45 and 47 years, respectively; *p* = 0.174), but were significantly different between the 2-year clearance and 2-year persistence groups (44 and 48 years, respectively; *p = *0.011 by a *t*-test) (Table 2). Mean values or distributions of body mass index, marital status, menopausal status, education level, income level, oral contraceptive use, smoking status, or cytological results (Pap smear test) did not differ between the clearance and persistence groups in t-test comparisons or chi-square distributions.

**Table 2:** General characteristics of the study subjects.

	One-year follow-up (n = 284)			Two-year follow-up (n = 122)		
	Clearance	Persistence	*p* 1)	Clearance	Persistence	*p*
Characteristics	(n = 148)	(n = 136)		(n = 66)	(n = 56)	
Age						
Mean (S.E.)	45.3 (0.66)	46.7 (0.74)	0.174	44.3 (0.95)	47.8 (0.99)	0.011
Body mass index						
Mean (S.E.)	22.5 (0.24)	22.2 (0.24)	0.505	22.5 (0.36)	21.5 (0.29)	0.034
Marital status (%)						
Single	2.1	4.6	0.232	3.2	1.9	0.675
Married	97.9	95.4		96.8	98.1	
Menopause (%)						
Pre	50.0	48.5	0.837	61.5	43.2	0.110
Post	50.0	51.5		38.5	56.8	
Number of children (%)						
None or one	11.9	10.8	0.939	17.3	13.0	0.764
Two	62.7	64.9		65.4	65.2	
≥Three	25.4	24.3		17.3	21.7	
Education (%)						
≤Middle school	13.3	16.4	0.123	12.9	11.5	0.355
High school	38.5	47.7		38.7	51.9	
≥University	48.2	35.9		48.4	36.5	
Income (won) (%)2)						
≤200 million	14.4	13.5	0.775	14.8	12.2	0.078
200–399 million	21.6	27.0		20.4	26.5	
400–699 million	41.6	40.5		35.2	51.0	
≥700 million	22.4	18.9		29.6	10.2	
Oral contraceptive use (%)						
Never	80.9	82.9	0.711	88.1	85.7	0.746
User (past/current)	19.1	17.1		11.9	12.3	
Smoking (%)						
Never	91.7	88.7	0.426	93.0	94.0	0.832
Smoker (past/current)	8.3	11.3		7.0	6.0	
Pap smear (%)						
Normal	81.1	80.9	0.997	75.8	71.4	0.864
ASCUS	11.5	11.8		15.1	17.9	
LSIL	7.4	7.3		9.1	10.7	

1) The chi-square test and t-test were used to analyze differences in the distribution of categorical and continuous variables, respectively.

2) The won-dollar exchange rate was approximately 1,280 won (per dollar) in 2002.

### Associations of viral load and alcohol consumption with persistent HPV infection

Median HPV loads were significantly higher in the 1-year and 2-year persistence groups than in the 1-year (*p*<0.001 by the Wilcoxon rank-sum test) and 2-year clearance (*p* = 0.001) groups, respectively (Table S1). As expected, the risks of 1-year persistence (multivariate odds ratio [mOR] 2.80, 95% confidence interval [CI] 1.64–4.78) and 2-year persistence (mOR 5.40, CI 2.25–12.9) were higher for a high HPV load than for a low HPV load. However, alcohol consumption (non-drinkers, drinkers), duration of alcohol drinking (<5 years, ≥5 years), and daily alcohol consumption (<15 g/day, ≥15 g/day) did not significantly affect the risk of 1-year or 2-year persistence.

### Risk of persistent HR-HPV infection according to the tertile of HPV load among drinkers and non-drinkers

Table 3 shows the ORs for 1-year (Table 3A) and 2-year persistence (Table 3B) according to the HPV load tertile among non-drinkers and drinkers. Among drinkers, high HPV loads were associated with high risks of 1-year (middle tertile: mOR 3.93, CI 1.96–7.89 and high tertile: mOR 4.09, CI 2.05–8.18; *p* for trend <0.001) and 2-year (middle tertile: mOR 2.55, CI 0.89–7.33 and high tertile: mOR 8.08, CI 2.36–27.6; *p* for trend <0.001) persistence. However, among non-drinkers, HPV load was not significantly associated with the risk of 1-year (middle tertile: mOR 1.02, CI 0.50–2.09 and high tertile: mOR 1.72, CI 0.85–3.46) or 2-year (middle tertile: mOR 0.82, CI 0.29–2.30 and high tertile: mOR 1.80, CI 0.70–4.61) persistence.

**Table 3:** Odds ratios and 95% confidence intervals for the risk of high risk-human papillomavirus (HR-HPV) infection among non-drinkers and drinkers according to the HR-HPV load tertile.

A.					
		*HR-HPV load tertile for the 1-year follow-up analysis* †			
Non-drinkers		T1 (n = 46)	T2 (n = 40)	T3 (n = 41)	*P* fortrend4)
HPV load (RLU/PC)	Range, Median1)	1.20–5.70, 1.80	6.09–107.10, 19.60	113.50–3085.80, 867.60	
	Crude	1 (ref.)	1.21 (0.61−2.38)	1.70 (0.86−3.36)	0.009
	Age-adj2)	1 (ref.)	1.13 (0.57−2.24)	1.68 (0.85−3.31)	0.003
	Multi-adj3)	1 (ref.)	1.02 (0.50−2.09)	1.72 (0.85−3.46)	0.003
**Drinkers**		**T1 (n = 52)**	**T2 (n = 51)**	**T3 (n = 54)**	***P*** ** for** **trend**
**HPV load (RLU/PC)**	**Range, Median**	**1.20–5.70, 1.53**	**6.09–107.10, 19.80**	**113.50–3085.80, 532.50**	
	Crude	1 (ref.)	3.34 (1.73−6.44)	3.08 (1.63−5.81)	<0.001
	Age-adj	1 (ref.)	3.79 (1.93−7.44)	3.78 (1.94−7.35)	<0.001
	Multi-adj	1 (ref.)	3.93 (1.96−7.89)	4.09 (2.05−8.18)	<0.001
**B.**					
		***HR-HPV load tertile in the 2-year follow-up analysis*** †			
**Non-drinkers**		**T1 (n = 24)**	**T2 (n = 17)**	**T3 (n = 18)**	***P*** ** for** **trend**
**HPV load (RLU/PC)**	**Range, Median**	**1.20–13.3, 2.82**	**13.56–164.60, 81.80**	**172.60–2934.20, 1269.15**	
	Crude	1 (ref.)	1.15 (0.40−3.25)	1.61 (0.58−4.48)	0.150
	Age-adj	1 (ref.)	0.84 (0.28−2.53)	1.59 (0.56−4.54)	0.125
	Multi-adj	1 (ref.)	0.84 (0.25−2.80)	1.33 (0.44−4.02)	0.559
**Drinkers**		**T1 (n = 17)**	**T2 (n = 23)**	**T3 (n = 23)**	***P*** ** for** **trend**
**HPV load (RLU/PC)**	**Range, Median**	**1.20–13.3, 4.00**	**13.56–164.60, 28.60**	**172.60–2934.20, 732.20**	
	Crude	1 (ref.)	1.77 (0.69−4.53)	3.04 (1.15−8.06)	0.005
	Age-adj	1 (ref.)	2.34 (0.86−6.39)	5.01 (1.69−14.9)	0.001
	Multi-adj	1 (ref.)	2.55 (0.89−7.33)	8.08 (2.36−27.6)	0.001

† HPV loads (relative light units [RLU]/positive control [PC]) in the 1- and 2-year follow-up studies were divided into three ranges by SAS software.

1) HPV load ranges and medians according to the T1, T2, and T3 tertiles of the two subject groups for non-drinkers and drinkers are presented.

2) Logistic regression analysis was performed after adjusting for age as a continuous variable. Risk estimates were calculated with the low viral load (T1) as the reference category.

3) Multivariate logistic regression analysis was performed after adjusting for age as a continuous variable and oral contraceptive use, menopausal status, smoking status, and number of children as categorical variables.

4) *p*-value for a OR linear trend according to the tertiles of HPV load in logistic regression model.

### Synergic effect of HR-HPV load and alcohol consumption on the risk of persistent HR-HPV infection

We found evidence of a synergistic effect of HR-HPV load and alcohol consumption on the risk of 1-year HPV persistence. The risk was higher for drinkers with a high HPV load (adjusted odds ratio [aOR] 3.69, CI 1.75–7.79; mOR 4.14, CI 1.89–9.05) than for non-drinkers with a low HPV load (Table S2, Table 4). On stratification analysis of HPV load according to alcohol consumption, the risk of 1-year persistence was similar in non-drinkers (mOR 3.10) and drinkers (mOR 2.83). The risk of 1-year persistence was higher for the combination of a high HPV load and drinking for ≥5 years (aOR 3.07, CI 1.44–6.57 and mOR 3.07, CI 1.40–6.75) than for a low HPV load and drinking for <5 years (Table S2, Table 3). The RERI and S for these combinations were 1.21 (CI −1.80–4.22) and 1.63 (CI 0.48–5.53), respectively (Table 4).

**Table 4:** Interaction between a high HR-HPV load and alcohol consumption on the risk of 1-year HR-HPV persistence.

	Low HR-HPV load1)		High HR-HPV load		*Multivariate OR for a* *high HPV load within* *the strata of alcohol* *consumption*	
	N w/wo	Multivariate OR	N w/wo	Multivariate OR		RERI^3)^
	persistence	(95% CI)2)	persistence	(95% CI)		S^4)^
**No alcohol consumption**	33/51	1 (ref.)	25/18	2.60 (1.19–5.68);	3.10 (1.31–7.36);	1.21 (−1.80–4.22);
				*p* = 0.017	*p = *0.010	*p = *0.433
**Alcohol consumption**	42/60	1.33 (0.71–2.50);	36/19	4.14 (1.89–9.05);	2.83 (1.37–5.84);	1.63 (0.48–5.53);
		*p = *0.381		*p*<0.001	*p = *0.005	*p = *0.434
*Multivariate OR for alcohol* *consumption within the* *strata of HPV load*		1.28 (0.66–2.46);		1.63 (0.61–4.39);		
		*p = *0.465		*p* = 0.330		
**Alcohol consumption** **for <5 years**	35/53	1 (ref.)	27/20	1.97 (0.98–3.98);	3.45 (1.46–8.45);	1.11 (−1.35–3.57);
				*p = *0.058	*p = *0.005	*p* = 0.376
**Alcohol consumption** **for ≥5 years**	27/40	0.99 (0.52–1.87);	14/27	3.07 (1.40–6.75);	2.93 (1.22–7.02);	2.16 (0.37–12.7);
		*p = *0.970		*p = *0.005	*p = *0.016	*p* = 0.395
*Multivariate OR for alcohol* *consumption for 5 years within* *the strata of HPV load*		1.17 (0.47−2.40);		1.48 (0.52–4.20);		
		*p = *0.676		*p = *0.465		
**Alcohol consumption** **of <15 g alcohol/day**	39/60	1 (ref.)	32/21	1.97 (1.03–3.80);	3.87 (1.73–8.63);	0.33 (−1.66–2.32);
				*p = *0.042	*p = *0.001	*p = *0.746
**Alcohol consumption** **of ≥15 g alcohol/day**	19/29	0.75 (0.37–1.53);	18/11	2.05 (0.87–4.83);	2.79 (0.93–8.40);	1.45 (0.14–14.8);
		*p = *0.428		*p = *0.100	*p = *0.067	*p = *0.751
*Multivariate OR for 15 g* *alcohol/day within the* *strata of HPV load*		1.09 (0.49–2.43);		1.17 (0.38–3.66);		
		*p = *0.840		*p = *0.786		

† N w/wo persistence, the number of subjects with/without persistence; OR, odds ratio.

1) HPV load was classified as low (<100 relative light units [RLU]/positive control [PC]) or high (≥100 RLU/PC).

2) Multivariate logistic regression analysis was performed with adjustment for age as a continuous variable and for menopausal status, oral contraceptive use, smoking status, and number of children as categorical variables. Risk estimates were calculated with no alcohol consumption, alcohol consumption for <5 years, or alcohol consumption <15 g/day and a low HPV load combination as reference categories.

3), 4) The relative excess risk due to interaction (RERI) and synergy index (S) were calculated as described by Rothman et al. RERI>0 and S>1.0 indicate a synergistic effect between HR-HPV load and alcohol consumption behaviors.

The risk of 2-year persistence was higher for the combination of a high HPV load and alcohol consumption (aOR 6.61, CI 2.09–20.9 and mOR 8.62, CI 2.46–30.2) than for a low HPV load and no alcohol consumption (Table 5, Table S3). On stratification analysis of HPV load according to alcohol consumption, the risk of 2-year persistence was 2.2-fold higher for drinkers (aOR 7.22) than for non-drinkers (aOR 3.29) (Table 5). In addition, the risk was higher for those with a high HPV load who had consumed alcohol for ≥5 years (aOR 5.72, CI 1.70–19.2 and mOR 6.40, CI 1.70–23.8) or had consumed ≥15 g alcohol/day (aOR 3.34, CI 1.03–10.7 and mOR 4.14, CI 1.18–14.6) (Table S3, Table 5). On stratification analysis of HPV load according to drinking behavior, the risk of 2-year persistence was 5- and 2-fold higher for drinking for ≥5 years versus <5 years (aOR 15.9 versus 3.30) and drinking ≥15 g alcohol/day versus <15 g alcohol/day (aOR 6.68 versus 3.23), respectively (Table 5). A strong synergistic effect of high HPV load and alcohol consumption on the risk of 2-year persistence was observed, with significant RERI (mOR 3.26, CI 1.42–5.09, *p*<0.001) and S values (mOR 2.38, CI 1.46–3.89, *p*<0.001).

**Table 5:** Effect of the interaction between a high HR-HPV load and alcohol consumption on the risk of 2-year HR-HPV persistence.

	Low HR-HPVload1)		High HR-HPVload		*Age-adjusted OR for* *a high HPV load within* *the strata of alcohol* *consumption*	
	N w/wo	Age-adjustedOR	N w/wo	Age-adjustedOR		RERI^3)^
	persistence	(95% CI)2)	persistence	(95% CI)		S^4)^
						
**No alcohol consumption**	11/23	1 (ref.)	15/10	3.37 (1.09–10.4);	3.29 (1.07–10.1);	3.26 (1.42–5.09);
				*p* = 0.035	*p* = 0.037	*p*<0.001
**Alcohol consumption**	10/23	0.99 (0.34–2.92);	20/10	6.61 (2.09–20.9);	7.22 (2.09–24.9);	2.38 (1.46–3.89);
		*p* = 0.988		*p* = 0.001	*p* = 0.002	*p*<0.001
*Age-adjusted OR for alcohol* *consumption within the* *strata of HPV load*		0.98 (0.34–2.86);		2.14 (0.62–7.45);		
		*p* = 0.975		*p* = 0.231		
**Alcohol consumption** **for <5 years**	12/23	1 (ref.)	17/11	2.57 (0.96–6.84);	3.30 (1.12–9.77);	3.66 (−1.74–9.06);
				*p* = 0.060	*p* = 0.031	*p* = 0.184
**Alcohol consumption** **for ≥5 years**	5/17	0.49 (0.15–1.59);	14/6	5.72 (1.70–19.2);	15.9 (2.71–93.4);	4.47 (0.25–78.3);
		*p* = 0.235		*p* = 0.005	*p* = 0.002	*p* = 0.305
*Age-adjusted OR for alcohol* *consumption within the* *strata of HPV load*		0.63 (0.18–2.20);		2.80 (0.66–11.9);		
		*p* = 0.466		*p* = 0.163		
**Alcohol consumption** **of <15 g alcohol/day**	13/26	1 (ref.)	32/21	2.40 (0.31–3.96);	3.23 (1.15–9.09);	0.84 (−3.32–4.50);
				*p* = 0.061	*p* = 0.026	*p* = 0.692
**Alcohol consumption** **of ≥15 g alcohol/day**	5/7	1.10 (0.31–3.96);	18/11	3.34 (1.03–10.7);	6.68 (0.83–53.9);	1.56 (0.18–13.7);
		*p* = 0.883		*p* = 0.044	*p* = 0.075	*p* = 0.687
*Age-adjusted OR for alcohol* *consumption within the* *strata of HPV load*		1.45 (0.38–5.63);		1.94 (0.45–8.32);		
		*p* = 0.589		*p* = 0.373		

† N w/wo persistence, the number of subjects with/without persistence; OR, odd ratio.

1) HPV load was classified as low (<100 relative light units [RLU]/positive control [PC]) or high (≥100 RLU/PC).

2) Logistic regression analysis was performed after adjustment for age as a continuous variable. Risk estimates were calculated with no alcohol consumption, alcohol consumption for <5 years, or alcohol consumption of <15 g/day and a low HPV load combination as reference categories.

3), 4) The relative excess risk due to interaction (RERI) and synergy index (S) were calculated as described by Rothman et al. RERI>0 and S>1.0 indicate a synergistic effect between HR-HPV load and alcohol consumption behaviors.

## Discussion

Our findings demonstrate an effect of the combination of HR-HPV load and alcohol consumption on the risk of HPV persistence in women with normal or low-grade cytological abnormalities; thus, a high viral load and alcohol behaviors may synergistically affect the risk of HR-HPV persistence. Furthermore, this synergistic effect is much stronger on longer-term HR-HPV infection.

Alcohol consumption is associated with an increased risk of HPV infection or acquisition [2], [17], but an association between alcohol consumption and persistent HPV infection has been rarely reported. Furthermore, to our knowledge, the combined effect of HPV load and alcohol consumption on the risk of persistent HPV infection has not been studied. In our previous study on other hospital subjects, women positive for HR-HPV with a high viral load who also consumed alcohol had markedly increased risks of CIN 1 (OR 19.1), but not CIN 2/3 [20]. The observations of the present study together with previous findings suggest that the synergistic effect of alcohol and HR-HPV load on the risk of virus-related cervical diseases might be exerted during the relatively early stages of cervical cancer pathogenesis. The synergistic effect of HR-HPV load and other risk factors on cervical pathogenesis has also been observed [18], [19]. One study reported a synergistic effect between smoking and high HPV-16 load on the risk of CIS, demonstrating that HPV-16-positive smokers with a high HPV load at the initial examination had a higher risk of CIS (OR 27.0) than HPV-16-negative smokers [18]. Another study showed that a high baseline HPV-16 or HPV-18 DNA load was associated with smoking in women with atypical squamous cells of undetermined significance or LSIL lesions [19]. In this study, we found that alcohol drinkers might have an increased risk of HR-HPV persistence even with a low viral load. Therefore, limiting alcohol consumption and cigarette smoking in women positive for HR-HPV infection with normal or low-grade cytological abnormalities might reduce the risk of persistent infection or progression to more severe stages.

The usefulness of clinical markers for HPV load has been debated. HPV-16 load has been reported to be associated with persistent infection [8], [9], cervical lesion severity (high-grade squamous intraepithelial lesions [HSIL]/LSIL) [10], [11], and pre-cancerous (CIN 3) or cancerous lesions of the cervix [8], [12]. However, HPV load has also been reported to be of limited use as a clinical parameter to discriminate between lesion grades [13], for HPV persistence prediction, or for the prediction of CIN development in women <30 years with normal cytology [14]. In this study, the total HR-HPV load at baseline was associated with a high risk of persistence compared to clearance. Our results are supported by several studies. High HPV load (>100 RLU/PC) was associated with HR-HPV persistence or HR-HPV clearance and lesion progression in women with normal cytology or low-grade cytological abnormalities but positive for HR-HPV DNA [24], [25]. Further, high HPV load (>400 RLU/PC) was a significant factor for recurrence after loop electrosurgical excision procedures for CIN 2 or 3 treatment [26], and the total HPV load or HPV-16 load was associated with the risk of HSIL or CIN 2 or 3 development [27].

Several mechanisms could explain the synergistic effects of HR-HPV load and alcohol consumption on viral persistence. Oxidative stress and HR-HPV can act synergistically to initiate and promote carcinogenesis. Viral infection, establishment of persistent infection, and viral integration are promoted by oxidative stress [28]. Numerous antioxidant enzymes and detoxifying pathways have been associated with HPV-transformed cells [29], [30]. Alcohol also activates and produces reactive oxygen species (ROS) through cytochrome p450 2E1 activation. ROS may elicit different host responses against HPV viral infection through highly variable amine and amine oxidase concentrations in the cervical mucus [31]. Furthermore, women with high ferritin levels are more likely to have persistent HR-HPV infection due to increased ROS levels [32]. Alcohol can also induce folate deficiency by blocking its absorption in the colon, leading to DNA hypomethylation [33]. High folate levels protect against the initiation of HPV-related dysplasia [34]. Thus, the combination of alcohol consumption and high HR-HPV load, a ROS-producer and potential risk factor, respectively, may increase the risk of viral persistence.

This study has several limitations. First, data could not be adjusted for sexual behavior or sexually transmitted diseases (STDs) such as chlamydia because the questionnaire did not include questions about these topics. However, the study subjects were mostly middle-aged women (mean age: 48 years), and a decline in sexual interest accompanied by a reduction in the frequency of sexual intercourse is often reported in aging women [35]. In a 2001 questionnaire study of Korean women, 88.6% and 70.5% reported an inactive sexual life in their forties and fifties, respectively, and those in their forties reported a peak sexual intercourse frequency of once a month [36]. Furthermore, investigation of sexual behaviors is difficult owing to the conservative Korean culture that limits truthful responses and the provision of information on sexual behaviors. A follow-up study conducted in Italy found no significant association between persistent HPV infection and a history of STDs; however, the association between *Chlamydia trachomatis* or Mycoplasma spp infection and HPV persistence could not be investigated because of the low detection rate of these organisms in the study population [37]. Korean women also had very low *C. trachomatis* prevalence, with an age-standardized prevalence of 4.3% [38]. Despite these expectations, the possibility of a relationship between sexual behavior and risk could not be excluded. Second, we could not determine whether persistent HR-HPV infections were of the same or new genotypes. In this study, HPV infection was detected using the HC2 test, which provides total viral load results, but no specific information on the 13 individual oncogenic HPV genotypes assayed. However, multiple-type HR-HPV infections in Korean women with normal pathology are reported to be low: the HR-HPV infection rate among women with normal cytology was 8.8%, and single and multiple-type infections among infected women were 8.3% and 0.5%, respectively [39]. In addition, 1-year HPV persistence detected by HC pooling of HPV genotypes is reported to be more sensitive for and predictive of CIN 3 than genotype-specific persistence based on a linear blot assay [40], [41]. Pooled detection of multiple oncogenic HPV genotypes can also minimize false negative results because multiple, concurrent noncausal types may be more readily detected than the single causal type [38]. Third, the relatively small numbers of subjects resulted in a limited statistical power especially in joint effect analysis and in multivariate analysis in the 2-year follow-up study. Future studies will have to be performed on a large scale to confirm our findings.

The HR-HPV load and alcohol consumption had a combined effect on viral persistence; the two factors synergistically increased the risk of persistent HR-HPV infection in women with normal or low-grade cytological abnormalities. Furthermore, this synergistic effect was much stronger on longer-term HR-HPV persistence. Limiting alcohol consumption might be important in preventing the development of cervical cancer in women with a high HR-HPV load.

## Supporting Information

Table S1 **Odds ratios and 95% confidence intervals of HR-HPV load and alcohol drinking for the risk of persistent high-risk human papillomavirus infection.** 1) P value was from Wilcoxon rank sum test after testing for normality. 2) HPV load value was classified as low (<100 relative light units [RLU]/positive control [PC]) or high (≥100 RLU/PC). 3) Multivariate logistic regression analysis was performed after adjusting for age as a continuous variable and for menopausal status, oral contraceptive use, smoking status and the number of children as categorical variables. The risks were estimated with the low HPV load, non-drinkers, <5 years, or <15 g alcohol/day as the reference categories.
(DOCX)

Table S2 **Interaction between high HR-HPV load and alcohol consumption on the risk of 1 year-HR-HPV persistence.** † N w/wo persistence, the number of subjects with/without persistence; OR, odds ratio. 1) HPV load value was classified as low (<100 relative light units [RLU]/positive control [PC]) or high (≥100 RLU/PC). 2) Logistic regression analysis was performed with adjustment for age as a continuous variable. The risk were estimated with no alcohol consumption, alcohol consumption for <5 years, or alcohol consumption of <15 g/day and a low HPV load as reference categories. 3), 4) The relative excess risk due to interaction (RERI) and synergy index(S) were calculated as described by Rothman et al. RERI>0 and S>1.0 indicate a synergistic effect between HR-HPV load and alcohol consumption behaviors.
(DOCX)

Table S3 **Interaction between high HR-HPV load and alcohol consumption on the risk of 2 year-HR-HPV persistence.** † N w/wo persistence, the number of subjects with/without persistence; OR, odds ratio. 1) HPV load value was classified as low (<100 relative light units [RLU]/positive control [PC]) or high (≥100 RLU/PC). 2) Multivariate logistic regression analysis was performed with adjustment for age as a continuous variable and for menopausal status and the number of children as categorical variables. The risk were estimated with no alcohol consumption, alcohol consumption for <5 years, or alcohol consumption of <15 g/day and low HPV load combination as reference categories. 3), 4) The relative excess risk due to interaction (RERI) and synergy index(S) were calculated as described by Rothman et al. The RERI>0 and S>1.0 indicate a synergistic effect between HR-HPV load and alcohol consumption behaviors.
(DOCX)
