# Correction: Synergistic Effect of Viral Load and Alcohol Consumption on the Risk of Persistent High-Risk Human Papillomavirus Infection

**DOI:** 10.1371/journal.pone.0124991

**Published:** 2015-04-13

**Authors:** Hea Young Oh, Sang-Soo Seo, Mi Kyung Kim, Dong Ock Lee, Youn Kyung Chung, Myong Cheol Lim, Joo-Young Kim, Chan Wha Lee, Sang-Yoon Park

There is an error in the second and third to last sentences of the “Subjects and groups” subsection of the Materials and Methods. The correct sentences are: Clearance was defined for both follow-up groups as HPV positivity at baseline with HPV negativity 1 year (n = 148) or 2 years later (n = 66) regardless of intermediate results. Persistence was defined as HPV positivity at baseline with HPV positivity at 1 (n = 136) or 2 years later (n = 56), regardless of intermediate results.

There are multiple errors in Table 5. Please see the corrected Table 5 here.

**Table 5:** Effect of the interaction between a high HR-HPV load and alcohol consumption on the risk of 2-year HR-HPV persistence.

	**Low HR-HPV load** ^1^ ^)^		**High HR-HPV load**		*Age-adjusted OR for a high HPV load within the strata of alcohol consumption*	
	N w/wo	Age-adjusted OR	N w/wo	Age-adjusted OR		RERI ^3^ ^)^
	persistence	(95% CI) ^2^ ^)^	persistence	(95% CI)		S ^4^ ^)^
**No alcohol consumption**	11/23	1 (ref.)	15/10	3.37 (1.09–10.4);	3.29 (1.07–10.1);	3.26 (1.42–5.09);
				*p* = 0.035	*p* = 0.037	*p* < 0.001
**Alcohol consumption**	10/23	0.99 (0.34–2.92);	20/10	6.61 (2.09–20.9);	7.22 (2.09–24.9);	2.38 (1.46–3.89);
		*p* = 0.988		*p* = 0.001	*p* = 0.002	*p* < 0.001
*Age-adjusted OR for alcohol consumption within the strata of HPV load*		0.98 (0.34–2.86);		2.14 (0.62–7.45);		
		*p* = 0.975		*p* = 0.231		
**Alcohol consumption for < 5 years**	12/23	1 (ref.)	17/11	2.57 (0.96–6.84);	3.30 (1.12–9.77);	3.66 (-1.74–9.06);
				*p* = 0.060	*p* = 0.031	*p* = 0.184
**Alcohol consumption for ≥ 5 years**	5/17	0.49 (0.15–1.59);	14/6	5.72 (1.70–19.2);	15.9 (2.71–93.4);	4.47 (0.25–78.3);
		*p* = 0.235		*p* = 0.005	*p* = 0.002	*p* = 0.305
*Age-adjusted OR for alcohol consumption within the strata of HPV load*		0.63 (0.18–2.20);		2.80 (0.66–11.9);		
		*p* = 0.466		*p* = 0.163		
**Alcohol consumption of < 15g alcohol/day**	13/26	1 (ref.)	18/12	2.40 (0.31–3.96);	3.23 (1.15–9.09);	0.84 (-3.32–4.50);
				*p* = 0.061	*p* = 0.026	*p* = 0.692
**Alcohol consumption of ≥ 15g alcohol/day**	5/7	1.10 (0.31–3.96);	10/6	3.34 (1.03–10.7);	6.68 (0.83–53.9);	1.56 (0.18–13.7);
		*p* = 0.883		*p* = 0.044	*p* = 0.075	*p* = 0.687
*Age-adjusted OR for alcohol consumption within the strata of HPV load*		1.45 (0.38–5.63);		1.94 (0.45–8.32);		
		*p* = 0.589		*p* = 0.373		

^†^ N w/wo persistence, the number of subjects with/without persistence; OR, odd ratio

^1)^ HPV load was classified as low (< 100 relative light units [RLU]/positive control [PC]) or high (≥ 100 RLU/PC).

^2)^ Logistic regression analysis was performed after adjustment for age as a continuous variable. Risk estimates were calculated with no alcohol consumption, alcohol consumption for < 5 years, or alcohol consumption of < 15 g/day and a low HPV load combination as reference categories.

^3)^ and ^4)^ The relative excess risk due to interaction (RERI) and synergy index (S) were calculated as described by Rothman et al. RERI > 0 and S > 1.0 indicate a synergistic effect between HR-HPV load and alcohol consumption behaviors.
