# Supplementary material for: Synergistic Effect of Viral Load and Alcohol Consumption on the Risk of Persistent High-Risk Human Papillomavirus Infection
Source: PLoS One. 2014 Aug 20;9(8):e104374. doi: 10.1371/journal.pone.0104374 (PMC4139267; doi:10.1371/journal.pone.0104374)
Supplement: Table S1 — Odds ratios and 95% confidence intervals of HR-HPV load and alcohol drinking for the risk of persistent high-risk human papillomavirus infection. 1) P value was from Wilcoxon rank sum test after testing for normality. 2) HPV load value was classified as low (<100 relative light units [RLU]/positive control [PC]) or high (≥100 RLU/PC). 3) Multivariate logistic regression analysis was performed after adjusting for age as a continuous variable and for menopausal status, oral contraceptive use, smoking status and the number of children as categorical variables. The risks were estimated with the low HPV load, non-drinkers, <5 years, or <15 g alcohol/day as the reference categories. (DOCX) [file pone.0104374.s001.docx]

**Table S1.** Odds ratios and 95% confidence intervals of HR-HPV load and alcohol drinking for the risk of persistent high-risk human papillomavirus infection

|  | One-year follow-up persistence (n=284) | | |  | Two-year follow-up persistence (n=122) | | |
| --- | --- | --- | --- | --- | --- | --- | --- |
|  | Clearance | Persistence | Multivariate odds ratios (95% CI) |  | Clearance | Persistence | Multivariate odds ratios (95% CI) |
| Characteristics | (n=148) | (n=136) |  |  | (n=66) | (n=56) |  |
| HPV load (Median) | 5.40 | 47.30 | p<0.001 ^1)^ |  | 17.90 | 136.55 | p=0.001 |
| HPV load group ^2)^ |  |  |  |  |  |  |  |
| Low (< 100 RLU/PC) | 111 (75.0) | 75 (55.2) | 1 (ref.) |  | 46 (69.7) | 21 (37.5) | 1 (ref.) |
| High (≥100 RLU/PC) | 37 (25.0) | 61 (44.9) | 2.80 (1.64−4.78) ^3)^ |  | 20 (30.3) | 35 (62.5) | 5.40 (2.25−12.9) |
| Alcohol drinking |  |  |  |  |  |  |  |
| Non-drinkers | 69 (46.6) | 58 (42.7) | 1 (ref.) |  | 33 (50.0) | 26 (46.4) | 1 (ref.) |
| Drinkers | 79 (53.4) | 78 (57.3) | 1.31 (0.79−2.18) |  | 33 (50.0) | 30 (53.6) | 1.48 (0.66−3.32) |
| Duration of drinking |  |  |  |  |  |  |  |
| Never, <5 years | 73 (57.5) | 62 (53.5) | 1 (ref.) |  | 34 (59.7) | 29 (60.4) | 1 (ref.) |
| ≥ 5 years | 54 (42.5) | 54 (46.5) | 1.28 (0.73−2.24) |  | 23 (40.3) | 19 (39.6) | 1.17 (0.45−3.01) |
| Drinking amount of alcohol |  |  |  |  |  |  |  |
| <15 g/day | 81 (66.9) | 71 (65.7) | 1 (ref.) |  | 38 (74.5) | 31 (67.4) | 1 (ref.) |
| ≥ 15 g/day | 40 (33.1) | 37 (34.3) | 1.01 (0.55−1.85) |  | 13 (25.5) | 15 (32.6) | 1.46 (0.53−4.02) |

1) P value was from Wilcoxon rank sum test after testing for normality.

2) HPV load value was classified as low (< 100 relative light units [RLU]/positive control [PC]) or high (≥ 100 RLU/PC).

3) Multivariate logistic regression analysis was performed after adjusting for age as a continuous variable and for menopausal status, oral contraceptive use, smoking status and the number of children as categorical variables. The risks were estimated with the low HPV load, non-drinkers, < 5 years, or < 15g alcohol/day as the reference categories.
