# Supplementary material for: Synergistic Effect of Viral Load and Alcohol Consumption on the Risk of Persistent High-Risk Human Papillomavirus Infection
Source: PLoS One. 2014 Aug 20;9(8):e104374. doi: 10.1371/journal.pone.0104374 (PMC4139267; doi:10.1371/journal.pone.0104374)
Supplement: Table S2 — Interaction between high HR-HPV load and alcohol consumption on the risk of 1 year-HR-HPV persistence. † N w/wo persistence, the number of subjects with/without persistence; OR, odds ratio. 1) HPV load value was classified as low (<100 relative light units [RLU]/positive control [PC]) or high (≥100 RLU/PC). 2) Logistic regression analysis was performed with adjustment for age as a continuous variable. The risk were estimated with no alcohol consumption, alcohol consumption for <5 years, or alcohol consumption of <15 g/day and a low HPV load as reference categories. 3), 4) The relative excess risk due to interaction (RERI) and synergy index(S) were calculated as described by Rothman et al. RERI>0 and S>1.0 indicate a synergistic effect between HR-HPV load and alcohol consumption behaviors. (DOCX) [file pone.0104374.s002.docx]

**Table S2.** Interaction between high HR-HPV load and alcohol consumption on the risk of 1year-HR-HPV persistence

|  | **Low HR-HPV load ^1)^** | |  | **High HR-HPV load** | |  | *Age-adj. OR for a high HPV load within the strata of alcohol consumption* |  |
| --- | --- | --- | --- | --- | --- | --- | --- | --- |
|  | N w/wo | Age-adjusted OR |  | N w/wo | Age-adjusted OR |  |  | RERI ^3)^ |
|  | persistence | (95% CI)^2)^ |  | persistence | (95% CI) |  |  | S ^4)^ |
|  |  |  |  |  |  |  |  |  |
| **No alcohol consumption** | 33/51 | 1 (ref.) |  | 25/18 | 2.39 (1.12–5.13); |  | 2.69 (1.22–5.96); | 1.06 (-1.55–3.67); |
|  |  |  |  |  | *p* = 0.025 |  | *p* = 0.015 | *p* = 0.426 |
| **Alcohol consumption** | 42/60 | 1.24 (0.67–2.27); |  | 36/19 | 3.69 (1.75–7.79); |  | 2.75 (1.38–5.50); | 1.65 (0.46–5.92); |
|  |  | *p* = 0.493 |  |  | *p* < 0.001 |  | *p* = 0.004 | *p* = 0.044 |
| *Age-adjusted OR for alcohol consumption within the strata of HPV load* |  | 1.19 (0.65–2.19); |  |  | 1.68 (0.71–3.99); |  |  |  |
|  |  | *p* = 0.574 |  |  | *p* = 0.242 |  |  |  |
|  |  |  |  |  |  |  |  |  |
| **Alcohol consumption for < 5 years** | 35/53 | 1 (ref.) |  | 27/20 | 1.97 (0.99–3.98); |  | 2.64 (1.22–5.69); | 1.11 (-1.35–3.57); |
|  |  |  |  |  | *p* = 0.054 |  | *p* = 0.014 | *p* = 0.378 |
| **Alcohol consumption for ≥ 5 years** | 27/40 | 1.01 (0.55–1.86); |  | 14/27 | 3.07 (1.44–6.57); |  | 2.84 (1.26–6.41); | 2.16 (0.37–12.7); |
|  |  | *p* = 0.984 |  |  | *p* = 0.004 |  | *p* = 0.012 | *p* = 0.393 |
| *Age-adjusted OR for alcohol consumption within the strata of HPV load* |  | 1.14 (0.58–2.24); |  |  | 1.74 (0.70–4.31); |  |  |  |
|  |  | *p* = 0.713 |  |  | *p* = 0.235 |  |  |  |
|  |  |  |  |  |  |  |  |  |
| **Alcohol consumption of < 15g alcohol/day** | 39/60 | 1 (ref.) |  | 32/21 | 2.05 (1.08–3.88); |  | 2.95 (1.43–6.09); | 0.41 (-1.78–2.59); |
|  |  |  |  |  | *p* = 0.029 |  | *p* = 0.004 | *p* = 0.715 |
| **Alcohol consumption of ≥ 15g alcohol/day** | 19/29 | 0.91 (0.47–1.77); |  | 18/11 | 2.36 (1.03–5.40); |  | 2.43 (0.94–6.29); | 1.43 (0.21–9.56); |
|  |  | *p* = 0.773 |  |  | *p* = 0.043 |  | *p* = 0.068 | *p* = 0.714 |
| *Age-adjusted OR for alcohol consumption within the strata of HPV load* |  | 1.11 (0.53–2.31); |  |  | 1.34 (0.50–3.57); |  |  |  |
|  |  | *p* = 0.787 |  |  | *p* = 0.560 |  |  |  |

† N w/wo persistence, the number of subjects with/without persistence; OR, odds ratio

1) HPV load value was classified as low (< 100 relative light units [RLU]/positive control [PC]) or high (≥ 100 RLU/PC).

2) Logistic regression analysis was performed with adjustment for age as a continuous variable. The risk were estimated with no alcohol consumption, alcohol consumption for < 5 years, or alcohol consumption of < 15g/day and a low HPV load as reference categories.

3), 4) The relative excess risk due to interaction (RERI) and synergy index (S) were calculated as described by Rothman et al. RERI > 0 and S > 1.0 indicate a synergistic effect between HR-HPV load and alcohol consumption behaviors.
