# Supplementary material for: Synergistic Effect of Viral Load and Alcohol Consumption on the Risk of Persistent High-Risk Human Papillomavirus Infection
Source: PLoS One. 2014 Aug 20;9(8):e104374. doi: 10.1371/journal.pone.0104374 (PMC4139267; doi:10.1371/journal.pone.0104374)
Supplement: Table S3 — Interaction between high HR-HPV load and alcohol consumption on the risk of 2 year-HR-HPV persistence. † N w/wo persistence, the number of subjects with/without persistence; OR, odds ratio. 1) HPV load value was classified as low (<100 relative light units [RLU]/positive control [PC]) or high (≥100 RLU/PC). 2) Multivariate logistic regression analysis was performed with adjustment for age as a continuous variable and for menopausal status and the number of children as categorical variables. The risk were estimated with no alcohol consumption, alcohol consumption for <5 years, or alcohol consumption of <15 g/day and low HPV load combination as reference categories. 3), 4) The relative excess risk due to interaction (RERI) and synergy index(S) were calculated as described by Rothman et al. The RERI>0 and S>1.0 indicate a synergistic effect between HR-HPV load and alcohol consumption behaviors. (DOCX) [file pone.0104374.s003.docx]

**Table S3.** Interaction between high HR-HPV load and alcohol consumption on the risk of 2 year-HR-HPV persistence

|  | **Low HR-HPV load ^1)^** | |  | **High HR-HPV load** | |  | *Multivariate OR for a high HPV load within the strata of alcohol consumption* |  |
| --- | --- | --- | --- | --- | --- | --- | --- | --- |
|  | N w/wo | Multivariate OR |  | N w/wo | Multivariate OR |  |  | RERI ^3)^ |
|  | persistence | (95% CI) ^2)^ |  | persistence | (95% CI) |  |  | S ^4)^ |
|  |  |  |  |  |  |  |  |  |
| **No alcohol consumption** | 11/23 | 1 (ref.) |  | 15/10 | 2.73 (0.81–9.16); |  | 2.68 (0.80–9.01); | 6.06 (-3.57–15.7); |
|  |  |  |  |  | *p* = 0.104 |  | *p* = 0.112 | *p =* 0.218 |
| **Alcohol consumption** | 10/23 | 0.84 (0.26–2.68); |  | 20/10 | 8.62 (2.46–30.2); |  | 10.9 (2.49–47.5); | 4.85 (0.55–43.3); |
|  |  | *p* = 0.769 |  |  | *p* < 0.001 |  | *p* = 0.002 | *p =* 0.157 |
| *Multivariate OR for alcohol consumption within the strata of HPV load* |  | 1.03 (0.33–3.22); |  |  | 2.78 (0.69–11.3); |  |  |  |
|  |  | *p* = 0.963 |  |  | *p* = 0.150 |  |  |  |
|  |  |  |  |  |  |  |  |  |
| **Alcohol consumption for < 5 years** | 12/23 | 1 (ref.) |  | 17/11 | 1.93 (0.66–5.65); |  | 3.55 (1.14–11.1); | 5.19 (-2.85–13.2); |
|  |  |  |  |  | *p* = 0.231 |  | *p* = 0.029 | *p* = 0.206 |
| **Alcohol consumption for ≥ 5 years** | 5/17 | 0.28 (0.75–1.04); |  | 14/6 | 6.40 (1.70–23.8); |  | 17.5 (2.43–126.1); | 25.8 (0.01–758871); |
|  |  | *p* = 0.057 |  |  | *p* = 0.006 |  | *p* = 0.005 | *p* = 0.536 |
| *Multivariate OR for alcohol consumption for 5 years within the strata of HPV load* |  | 0.59 (0.15-2.34); |  |  | 3.98 (0.77–20.5); |  |  |  |
|  |  | *p* = 0.457 |  |  | *p* = 0.099 |  |  |  |
|  |  |  |  |  |  |  |  |  |
| **Alcohol consumption of < 15g alcohol/day** | 13/26 | 1 (ref.) |  | 32/21 | 2.30 (0.88–6.02); |  | 3.40 (1.18–9.80); | 1.97 (-3.18–7.10); |
|  |  |  |  |  | *p* = 0.091 |  | *p* = 0.023 | *p* = 0.454 |
| **Alcohol consumption of ≥ 15g alcohol/day** | 5/7 | 0.88 (0.21–3.65); |  | 18/11 | 4.14 (1.18–14.6); |  | 4.15 (0.43–40.4); | 2.67 (0.25–14.8); |
|  |  | *p* = 0.861 |  |  | *p* = 0.027 |  | *p* = 0.220 | *p* = 0.437 |
| *Multivariate OR for 15g alcohol/day within the strata of HPV load* |  | 0.90 (0.21–3.91); |  |  | 2.10 (0.44–10.1); |  |  |  |
|  |  | *p* = 0.892 |  |  | *p* = 0.355 |  |  |  |

† N w/wo persistence, the number of subjects with/without persistence; OR, odds ratio

1) HPV load value was classified as low (< 100 relative light units [RLU]/positive control [PC]) or high (≥ 100 RLU/PC).

2) Multivariate logistic regression analysis was performed with adjustment for age as a continuous variable and for menopausal status and the number of children as categorical variables. The risk were estimated with no alcohol consumption, alcohol consumption for < 5 years, or alcohol consumption of < 15g/day and low HPV load combination as reference categories.

3), 4) The relative excess risk due to interaction (RERI) and synergy index (S) were calculated as described by Rothman et al. The RERI > 0 and S > 1.0 indicate a synergistic effect between HR-HPV load and alcohol consumption behaviors.
